# Bis(5,5,7,12,12,14-hexa­methyl-1,4,8,11-tetra­azacyclo­tetra­decane-κ^4^ *N*)(μ-l-mal­ato-κ^4^ *O* ^1^,*O* ^2^:*O* ^4^,*O* ^4′^)dinickel(II) bis(perchlorate) monohydrate

**DOI:** 10.1107/S1600536809020662

**Published:** 2009-06-06

**Authors:** Guang-Chuan Ou, Qiang Zhou, Seik Weng Ng

**Affiliations:** a Department of Biology and Chemistry, Hunan University of Science and Engineering, Yongzhou, Hunan 425100, People’s Republic of China; b Department of Chemistry, University of Malaya, 50603 Kuala Lumpur, Malaysia

## Abstract

In the crystal structure of the title dinuclear compound, [Ni_2_(C_4_H_4_O_5_)(C_16_H_36_N_4_)_2_](ClO_4_)_2_·H_2_O, the bridg­ing di­car­box­yl­ate dianion *O*,*O*′-chelates to two Ni atoms, both of which are also chelated by the N-macrocylic ligand. The Ni atoms exhibit a distorted octa­hedral coordination. N—H⋯O and O—H⋯O hydrogen bonds link the cations and the uncoordinated water mol­ecules into a layer structure; the perchlorate anions occupy the space between adjacent layers, and are only weakly linked to the layers. One of the perchlorate anions is disordered over two sets of sites in a 3:2 ratio.

## Related literature

For the nickel phthalate perchlorate hydrate derivative of the macrocycle, see: Ou & Zhang (2009 ▶).
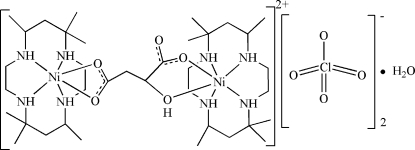


## Experimental

### 

#### Crystal data

[Ni_2_(C_4_H_4_O_5_)(C_16_H_36_N_4_)_2_](ClO_4_)_2_·H_2_O
*M* *_r_* = 1035.38
Orthorhombic, 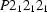

*a* = 13.562 (2) Å
*b* = 18.984 (2) Å
*c* = 19.350 (3) Å
*V* = 4982 (1) Å^3^
*Z* = 4
Mo *K*α radiation
μ = 0.93 mm^−1^
*T* = 293 K
0.48 × 0.45 × 0.34 mm

#### Data collection

Bruker SMART area-detector diffractometer
Absorption correction: multi-scan (*SADABS*; Sheldrick, 1996 ▶) *T*_min_ = 0.605, *T*_max_ = 0.730
42585 measured reflections
10997 independent reflections
7769 reflections with *I* > 2σ(*I*)
*R*_int_ = 0.043

#### Refinement

*R*[*F* ^2^ > 2σ(*F* ^2^)] = 0.043
*wR*(*F* ^2^) = 0.123
*S* = 1.03
10997 reflections
647 parameters
79 restraints
H atoms treated by a mixture of independent and constrained refinement
Δρ_max_ = 0.41 e Å^−3^
Δρ_min_ = −0.38 e Å^−3^
Absolute structure: Flack (1983 ▶), 4936 Friedel pairs
Flack parameter: −0.01 (2)

### 

Data collection: *SMART* (Bruker, 1999 ▶); cell refinement: *SAINT-Plus* (Bruker, 1999 ▶); data reduction: *SAINT-Plus*; program(s) used to solve structure: *SHELXS97* (Sheldrick, 2008 ▶); program(s) used to refine structure: *SHELXL97* (Sheldrick, 2008 ▶); molecular graphics: *X-SEED* (Barbour, 2001 ▶); software used to prepare material for publication: *publCIF* (Westrip, 2009 ▶).

## Supplementary Material

Crystal structure: contains datablocks I, global. DOI: 10.1107/S1600536809020662/xu2535sup1.cif

Structure factors: contains datablocks I. DOI: 10.1107/S1600536809020662/xu2535Isup2.hkl

Additional supplementary materials:  crystallographic information; 3D view; checkCIF report

## Figures and Tables

**Table 1:** Hydrogen-bond geometry (Å, °)

*D*—H⋯*A*	*D*—H	H⋯*A*	*D*⋯*A*	*D*—H⋯*A*
N2—H2⋯O6^i^	0.84 (5)	2.41 (2)	3.230 (7)	165 (6)
N5—H5⋯O2^ii^	0.85 (5)	2.14 (3)	2.940 (6)	158 (6)
N7—H7⋯O2^ii^	0.85 (2)	2.06 (2)	2.869 (5)	160 (6)
O1w—H11⋯O5	0.86 (9)	2.09 (7)	2.862 (9)	149 (12)
O1w—H12⋯O6	0.86 (9)	2.05 (8)	2.784 (12)	143 (13)

Symmetry codes: (i) 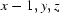; (ii) 
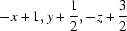
.

**Fig. 1.**
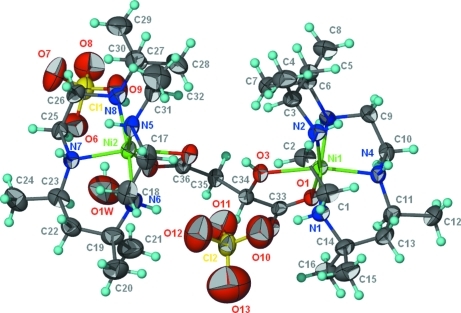
Thermal ellipsoid plot (Barbour, 2001) of [Ni2(C16H36N4)2(C4H4O5)]2+2(ClO4)-.H2O showing displacement ellipsoids of the dinculear salt hydrate at the 50% probability level. H-atoms are drawn as spheres of arbitrary radii. The disorder in the perchlorate is not shown.

## supplementary crystallographic information

### Experimental

L-Malic acid (0.13 g, 1 mmol) was neutralized with sodium hydroxide (0.08 g, 2 mmol) in water (10 ml). To this solution was added (5,5,7,12,12,14 -hexamethyl-1,4,8,11-tetraazacyclotetradecane)nickel perchlorate (0.54 g, 1 mmol) dissolved in acetonitrile (10 ml). The solution was left to stand at room temperature; blue crystals formed after several weeks.

### Refinement

Carbon bound H-atoms were positioned geometrically and refined using the riding model, with C—H = 0.93 to 0.98 Å and *U*(H) set to 1.2–1.5 *U*_eq_(C).

H atoms attached to the N– and O-atoms were located in difference Fourier maps and were refined with a distance restraint of N–H = O–H = 0.85±0.01 Å; their temperature factors were restrained to 1.5 times *U*_eq_(N,*O*).

One of the two anions is disordered over two positions. The Cl–O distance was restrained to 1.45±0.01 Å and the O···O distance to 2.37±0.02 Å. The anisotropic temperature factors of the oxygen atoms were restrained to be nearly isotropic. The disorder refined to a 3:2 ratio.

### Crystal data

**Table:**

[Ni_2_(C_4_H_4_O_5_)(C_16_H_36_N_4_)_2_](ClO_4_)_2_·H_2_O	*F*(000) = 2208
*M**_r_* = 1035.38	*D*_x_ = 1.381 Mg m^−^^3^
Orthorhombic, *P*2_1_2_1_2_1_	Mo *K*α radiation, λ = 0.71073 Å
Hall symbol: P 2ac 2ab	Cell parameters from 1019 reflections
*a* = 13.562 (2) Å	θ = 2.8–22.5°
*b* = 18.984 (2) Å	µ = 0.93 mm^−^^1^
*c* = 19.350 (3) Å	*T* = 293 K
*V* = 4982 (1) Å^3^	Block, blue
*Z* = 4	0.48 × 0.45 × 0.34 mm

### Data collection

**Table:**

Bruker SMART area-detector diffractometer	10997 independent reflections
Radiation source: fine-focus sealed tube	7769 reflections with *I* > 2σ(*I*)
graphite	*R*_int_ = 0.043
φ and ω scans	θ_max_ = 27.2°, θ_min_ = 1.8°
Absorption correction: multi-scan (*SADABS*; Sheldrick, 1996)	*h* = −17→17
*T*_min_ = 0.605, *T*_max_ = 0.730	*k* = −24→24
42585 measured reflections	*l* = −24→24

### Refinement

**Table:**

Refinement on *F*^2^	Secondary atom site location: difference Fourier map
Least-squares matrix: full	Hydrogen site location: inferred from neighbouring sites
*R*[*F*^2^ > 2σ(*F*^2^)] = 0.043	H atoms treated by a mixture of independent and constrained refinement
*wR*(*F*^2^) = 0.123	*w* = 1/[σ^2^(*F*_o_^2^) + (0.066*P*)^2^ + 1.0201*P*] where *P* = (*F*_o_^2^ + 2*F*_c_^2^)/3
*S* = 1.03	(Δ/σ)_max_ = 0.001
10997 reflections	Δρ_max_ = 0.41 e Å^−^^3^
647 parameters	Δρ_min_ = −0.38 e Å^−^^3^
79 restraints	Absolute structure: Flack (Flack, 1983), 4936 Friedel pairs
Primary atom site location: structure-invariant direct methods	Flack parameter: −0.01 (2)

### Fractional atomic coordinates and isotropic or equivalent isotropic displacement parameters (Å^2^)

**Table:**

	*x*	*y*	*z*	*U*_iso_*/*U*_eq_	Occ. (<1)
Ni1	0.12840 (5)	0.77230 (3)	0.82955 (3)	0.03650 (16)	
Ni2	0.54780 (5)	0.96584 (3)	0.76686 (3)	0.04267 (17)	
Cl1	0.82095 (12)	0.87472 (8)	0.96151 (9)	0.0635 (4)	
Cl2	0.2212 (4)	0.9094 (3)	0.5860 (2)	0.0694 (15)	0.603 (15)
Cl2'	0.2343 (12)	0.8981 (10)	0.5917 (8)	0.184 (9)	0.397 (15)
O1	0.2141 (2)	0.68426 (16)	0.81902 (18)	0.0410 (8)	
O2	0.3574 (3)	0.63875 (18)	0.7861 (2)	0.0589 (11)	
O3	0.2686 (2)	0.81315 (17)	0.79792 (18)	0.0430 (8)	
H3O	0.2801	0.8566	0.7964	0.052*	
O4	0.4208 (3)	0.89861 (18)	0.7931 (2)	0.0483 (9)	
O5	0.5716 (3)	0.85639 (19)	0.7953 (2)	0.0520 (9)	
O6	0.8605 (4)	0.8321 (3)	0.9084 (3)	0.1028 (18)	
O7	0.8598 (5)	0.9427 (3)	0.9528 (4)	0.120 (2)	
O8	0.8508 (5)	0.8471 (4)	1.0255 (3)	0.121 (2)	
O9	0.7173 (3)	0.8767 (3)	0.9558 (3)	0.0895 (16)	
O10	0.1180 (7)	0.9240 (9)	0.6026 (8)	0.145 (6)	0.603 (15)
O11	0.2768 (10)	0.8832 (8)	0.6437 (6)	0.130 (5)	0.603 (15)
O12	0.2533 (11)	0.9786 (6)	0.5709 (8)	0.159 (6)	0.603 (15)
O13	0.2280 (19)	0.8633 (11)	0.5287 (9)	0.254 (12)	0.603 (15)
O10'	0.237 (3)	0.9458 (18)	0.6497 (14)	0.29 (2)	0.397 (15)
O11'	0.2788 (14)	0.9246 (12)	0.5304 (10)	0.131 (8)	0.397 (15)
O12'	0.281 (2)	0.8324 (15)	0.6114 (19)	0.30 (2)	0.397 (15)
O13'	0.1331 (14)	0.8814 (14)	0.5764 (12)	0.153 (9)	0.397 (15)
O1W	0.7584 (7)	0.7864 (6)	0.7920 (5)	0.147 (3)	
H11	0.701 (5)	0.800 (8)	0.778 (7)	0.221*	
H12	0.762 (11)	0.804 (8)	0.833 (3)	0.221*	
N1	0.0790 (3)	0.7848 (2)	0.7234 (2)	0.0475 (10)	
H1	0.132 (3)	0.796 (3)	0.703 (3)	0.071*	
N2	0.0713 (3)	0.8748 (2)	0.8429 (2)	0.0434 (10)	
H2	0.015 (2)	0.872 (3)	0.861 (3)	0.065*	
N3	0.1520 (3)	0.7656 (2)	0.9405 (2)	0.0411 (10)	
H3	0.200 (5)	0.736 (3)	0.948 (3)	0.062*	
N4	0.0065 (3)	0.7076 (2)	0.8493 (2)	0.0441 (10)	
H4	−0.042 (3)	0.735 (3)	0.851 (3)	0.066*	
N5	0.4683 (3)	1.0599 (2)	0.7605 (2)	0.0481 (11)	
H5	0.508 (4)	1.092 (2)	0.748 (3)	0.072*	
N6	0.5049 (4)	0.9492 (3)	0.6629 (2)	0.0558 (12)	
H6	0.454 (3)	0.922 (3)	0.661 (4)	0.084*	
N7	0.6895 (3)	0.9928 (2)	0.7340 (2)	0.0421 (9)	
H7	0.690 (4)	1.0362 (10)	0.723 (3)	0.063*	
N8	0.5986 (3)	0.9947 (2)	0.8667 (2)	0.0456 (10)	
H8	0.610 (5)	0.9529 (14)	0.880 (3)	0.068*	
C1	0.0142 (5)	0.8480 (3)	0.7268 (3)	0.0592 (15)	
H1A	−0.0499	0.8349	0.7450	0.071*	
H1B	0.0048	0.8670	0.6807	0.071*	
C2	0.0603 (5)	0.9028 (3)	0.7724 (3)	0.0547 (14)	
H2A	0.1244	0.9158	0.7543	0.066*	
H2B	0.0192	0.9446	0.7733	0.066*	
C3	0.1289 (5)	0.9216 (3)	0.8894 (3)	0.0486 (12)	
H3a	0.1979	0.9196	0.8745	0.058*	
C4	0.0963 (6)	0.9980 (3)	0.8861 (4)	0.0730 (19)	
H4A	0.1147	1.0176	0.8422	0.109*	
H4B	0.1276	1.0241	0.9225	0.109*	
H4C	0.0260	1.0006	0.8914	0.109*	
C5	0.1246 (4)	0.8945 (3)	0.9628 (3)	0.0496 (13)	
H5A	0.0560	0.8849	0.9735	0.059*	
H5B	0.1454	0.9325	0.9930	0.059*	
C6	0.1849 (4)	0.8283 (3)	0.9827 (3)	0.0475 (13)	
C7	0.2919 (4)	0.8393 (4)	0.9707 (3)	0.0617 (16)	
H7A	0.3030	0.8502	0.9229	0.093*	
H7B	0.3272	0.7971	0.9826	0.093*	
H7C	0.3149	0.8775	0.9989	0.093*	
C8	0.1688 (5)	0.8155 (4)	1.0609 (3)	0.0663 (17)	
H8A	0.1966	0.7708	1.0736	0.099*	
H8B	0.0995	0.8154	1.0708	0.099*	
H8C	0.2004	0.8522	1.0868	0.099*	
C9	0.0614 (4)	0.7314 (3)	0.9669 (3)	0.0479 (12)	
H9A	0.0098	0.7663	0.9724	0.057*	
H9B	0.0744	0.7104	1.0117	0.057*	
C10	0.0286 (4)	0.6755 (3)	0.9168 (3)	0.0476 (13)	
H10A	0.0802	0.6405	0.9116	0.057*	
H10B	−0.0298	0.6521	0.9345	0.057*	
C11	−0.0160 (4)	0.6528 (3)	0.7949 (3)	0.0508 (13)	
H11A	0.0440	0.6251	0.7871	0.061*	
C12	−0.0966 (6)	0.6027 (4)	0.8186 (4)	0.081 (2)	
H12A	−0.1108	0.5697	0.7824	0.121*	
H12B	−0.1550	0.6291	0.8294	0.121*	
H12C	−0.0748	0.5778	0.8590	0.121*	
C13	−0.0437 (4)	0.6878 (3)	0.7277 (3)	0.0582 (14)	
H13A	−0.0940	0.7225	0.7380	0.070*	
H13B	−0.0745	0.6522	0.6989	0.070*	
C14	0.0346 (4)	0.7242 (3)	0.6839 (3)	0.0547 (14)	
C15	−0.0159 (6)	0.7498 (4)	0.6154 (3)	0.081 (2)	
H15A	−0.0742	0.7763	0.6264	0.122*	
H15B	−0.0336	0.7098	0.5878	0.122*	
H15C	0.0291	0.7791	0.5901	0.122*	
C16	0.1187 (5)	0.6740 (3)	0.6648 (3)	0.0664 (16)	
H16A	0.1491	0.6565	0.7061	0.100*	
H16B	0.1667	0.6988	0.6377	0.100*	
H16C	0.0929	0.6353	0.6385	0.100*	
C17	0.4060 (5)	1.0505 (4)	0.6974 (3)	0.0677 (18)	
H17A	0.3508	1.0198	0.7079	0.081*	
H17B	0.3801	1.0958	0.6828	0.081*	
C18	0.4658 (5)	1.0195 (3)	0.6410 (3)	0.0686 (18)	
H18A	0.5202	1.0506	0.6299	0.082*	
H18B	0.4255	1.0140	0.6000	0.082*	
C19	0.5745 (6)	0.9148 (4)	0.6122 (3)	0.073 (2)	
C20	0.5342 (7)	0.9181 (5)	0.5379 (4)	0.103 (3)	
H20A	0.5351	0.9660	0.5219	0.154*	
H20B	0.4677	0.9007	0.5371	0.154*	
H20C	0.5746	0.8897	0.5082	0.154*	
C21	0.5837 (7)	0.8374 (4)	0.6313 (4)	0.096 (3)	
H21A	0.6043	0.8333	0.6786	0.144*	
H21B	0.6316	0.8153	0.6019	0.144*	
H21C	0.5210	0.8146	0.6255	0.144*	
C22	0.6739 (5)	0.9521 (4)	0.6130 (3)	0.0643 (16)	
H22A	0.7134	0.9318	0.5763	0.077*	
H22B	0.6623	1.0009	0.6007	0.077*	
C23	0.7364 (5)	0.9517 (3)	0.6780 (3)	0.0576 (15)	
H23	0.7438	0.9029	0.6936	0.069*	
C24	0.8386 (5)	0.9808 (5)	0.6604 (4)	0.083 (2)	
H24A	0.8750	0.9886	0.7022	0.124*	
H24B	0.8317	1.0246	0.6359	0.124*	
H24C	0.8732	0.9476	0.6319	0.124*	
C25	0.7485 (4)	0.9935 (3)	0.7985 (3)	0.0495 (13)	
H25A	0.8109	1.0169	0.7903	0.059*	
H25B	0.7621	0.9455	0.8129	0.059*	
C26	0.6935 (4)	1.0309 (3)	0.8540 (3)	0.0519 (13)	
H26A	0.7323	1.0314	0.8961	0.062*	
H26B	0.6814	1.0792	0.8402	0.062*	
C27	0.5318 (4)	1.0307 (3)	0.9167 (3)	0.0549 (13)	
C28	0.4578 (5)	0.9774 (3)	0.9420 (3)	0.0734 (18)	
H28A	0.4230	0.9579	0.9032	0.110*	
H28B	0.4118	1.0000	0.9725	0.110*	
H28C	0.4914	0.9405	0.9663	0.110*	
C29	0.5892 (6)	1.0587 (4)	0.9801 (4)	0.085 (2)	
H29A	0.6321	1.0963	0.9659	0.127*	
H29B	0.6276	1.0213	0.9998	0.127*	
H29C	0.5434	1.0760	1.0139	0.127*	
C30	0.4802 (5)	1.0937 (3)	0.8827 (3)	0.0613 (16)	
H30A	0.4424	1.1177	0.9182	0.074*	
H30B	0.5308	1.1262	0.8673	0.074*	
C31	0.4119 (4)	1.0802 (3)	0.8222 (3)	0.0563 (15)	
H31	0.3682	1.0410	0.8345	0.068*	
C32	0.3483 (6)	1.1456 (5)	0.8105 (5)	0.098 (3)	
H32A	0.3125	1.1407	0.7679	0.147*	
H32B	0.3898	1.1865	0.8081	0.147*	
H32C	0.3026	1.1507	0.8480	0.147*	
C33	0.3003 (4)	0.6883 (2)	0.7964 (3)	0.0396 (11)	
C34	0.3416 (3)	0.7616 (2)	0.7795 (3)	0.0398 (11)	
H34	0.3525	0.7643	0.7295	0.048*	
C35	0.4385 (3)	0.7751 (3)	0.8153 (3)	0.0430 (11)	
H35A	0.4858	0.7399	0.8006	0.052*	
H35B	0.4293	0.7700	0.8648	0.052*	
C36	0.4800 (4)	0.8474 (3)	0.8008 (3)	0.0435 (12)	

### Atomic displacement parameters (Å^2^)

**Table:**

	*U*^11^	*U*^22^	*U*^33^	*U*^12^	*U*^13^	*U*^23^
Ni1	0.0370 (3)	0.0302 (3)	0.0422 (3)	0.0003 (3)	0.0024 (3)	−0.0010 (3)
Ni2	0.0445 (4)	0.0362 (3)	0.0472 (4)	−0.0054 (3)	−0.0047 (3)	0.0082 (3)
Cl1	0.0550 (9)	0.0606 (9)	0.0750 (10)	0.0010 (7)	−0.0042 (8)	0.0111 (8)
Cl2	0.065 (3)	0.087 (3)	0.056 (2)	−0.024 (2)	−0.0080 (18)	0.0161 (18)
Cl2'	0.131 (11)	0.207 (17)	0.213 (18)	−0.036 (10)	−0.047 (11)	0.041 (14)
O1	0.0395 (19)	0.0307 (16)	0.053 (2)	0.0006 (14)	0.0074 (16)	−0.0038 (15)
O2	0.048 (2)	0.0317 (18)	0.097 (3)	0.0023 (17)	0.015 (2)	−0.0147 (19)
O3	0.0387 (18)	0.0281 (16)	0.062 (2)	0.0007 (14)	0.0060 (16)	0.0005 (15)
O4	0.047 (2)	0.0347 (18)	0.063 (2)	−0.0022 (16)	−0.0028 (17)	0.0084 (16)
O5	0.044 (2)	0.042 (2)	0.070 (2)	−0.0066 (16)	0.0000 (18)	0.0012 (18)
O6	0.071 (3)	0.131 (5)	0.107 (4)	0.019 (3)	0.005 (3)	−0.024 (4)
O7	0.099 (4)	0.085 (4)	0.175 (6)	−0.032 (3)	−0.040 (4)	0.030 (4)
O8	0.140 (6)	0.131 (5)	0.090 (4)	0.010 (5)	−0.022 (4)	0.038 (4)
O9	0.054 (3)	0.081 (3)	0.133 (5)	0.011 (2)	0.011 (3)	0.016 (3)
O10	0.139 (9)	0.161 (10)	0.135 (9)	−0.027 (8)	−0.001 (7)	0.032 (8)
O11	0.141 (9)	0.141 (9)	0.107 (8)	−0.011 (7)	−0.045 (7)	0.047 (7)
O12	0.152 (9)	0.151 (10)	0.175 (11)	−0.035 (8)	−0.022 (8)	0.048 (9)
O13	0.260 (16)	0.253 (16)	0.249 (15)	0.024 (12)	−0.024 (12)	−0.043 (11)
O10'	0.29 (3)	0.29 (3)	0.30 (3)	−0.018 (13)	0.009 (13)	−0.009 (13)
O11'	0.134 (11)	0.146 (12)	0.114 (11)	−0.023 (9)	0.038 (9)	0.044 (9)
O12'	0.31 (3)	0.30 (3)	0.29 (3)	0.013 (13)	0.002 (13)	0.007 (13)
O13'	0.144 (13)	0.164 (13)	0.152 (13)	−0.055 (11)	0.002 (10)	0.011 (10)
O1W	0.132 (6)	0.163 (8)	0.147 (7)	0.045 (6)	0.011 (5)	−0.018 (6)
N1	0.048 (3)	0.049 (3)	0.045 (3)	−0.001 (2)	−0.004 (2)	−0.002 (2)
N2	0.048 (3)	0.0305 (19)	0.051 (3)	0.0031 (18)	0.000 (2)	−0.0006 (18)
N3	0.040 (2)	0.042 (2)	0.041 (2)	0.0061 (19)	0.0043 (18)	−0.0029 (18)
N4	0.039 (2)	0.037 (2)	0.056 (3)	−0.0009 (18)	0.005 (2)	−0.0022 (19)
N5	0.041 (2)	0.041 (2)	0.062 (3)	−0.0029 (18)	−0.004 (2)	0.013 (2)
N6	0.066 (3)	0.058 (3)	0.043 (2)	−0.023 (2)	−0.010 (2)	0.009 (2)
N7	0.045 (2)	0.034 (2)	0.047 (2)	−0.0017 (18)	0.001 (2)	0.005 (2)
N8	0.051 (3)	0.038 (2)	0.048 (3)	0.000 (2)	−0.003 (2)	0.003 (2)
C1	0.075 (4)	0.047 (3)	0.056 (3)	0.010 (3)	−0.013 (3)	0.004 (3)
C2	0.070 (4)	0.040 (3)	0.054 (3)	0.013 (3)	−0.004 (3)	0.007 (2)
C3	0.058 (3)	0.037 (3)	0.051 (3)	0.002 (3)	0.002 (3)	−0.007 (2)
C4	0.111 (6)	0.040 (3)	0.067 (4)	0.002 (3)	0.005 (4)	−0.011 (3)
C5	0.054 (3)	0.047 (3)	0.048 (3)	0.003 (3)	0.004 (3)	−0.011 (2)
C6	0.058 (3)	0.047 (3)	0.038 (3)	−0.002 (3)	−0.003 (2)	−0.009 (2)
C7	0.053 (3)	0.074 (4)	0.059 (4)	−0.008 (3)	−0.002 (3)	−0.011 (3)
C8	0.090 (5)	0.063 (4)	0.046 (3)	0.010 (3)	0.002 (3)	−0.004 (3)
C9	0.044 (3)	0.050 (3)	0.049 (3)	0.006 (3)	0.008 (2)	0.000 (2)
C10	0.044 (3)	0.042 (3)	0.057 (3)	−0.002 (2)	0.011 (3)	0.006 (2)
C11	0.049 (3)	0.040 (3)	0.062 (3)	−0.005 (2)	0.001 (3)	−0.009 (3)
C12	0.085 (5)	0.071 (4)	0.086 (5)	−0.036 (4)	0.012 (4)	−0.020 (4)
C13	0.052 (3)	0.054 (3)	0.068 (4)	−0.006 (3)	−0.009 (3)	−0.015 (3)
C14	0.057 (3)	0.056 (3)	0.051 (3)	−0.004 (3)	−0.008 (3)	−0.011 (3)
C15	0.107 (6)	0.076 (5)	0.061 (4)	−0.005 (4)	−0.021 (4)	−0.008 (3)
C16	0.077 (4)	0.062 (4)	0.059 (4)	0.002 (3)	0.006 (4)	−0.019 (3)
C17	0.056 (4)	0.077 (4)	0.070 (4)	0.001 (3)	−0.020 (3)	0.026 (4)
C18	0.070 (4)	0.073 (4)	0.063 (4)	−0.011 (3)	−0.019 (3)	0.029 (3)
C19	0.105 (6)	0.072 (4)	0.044 (3)	−0.015 (4)	0.003 (4)	0.000 (3)
C20	0.124 (7)	0.134 (7)	0.050 (4)	−0.035 (6)	−0.009 (4)	−0.011 (4)
C21	0.143 (8)	0.068 (5)	0.076 (5)	−0.019 (5)	0.015 (5)	−0.012 (4)
C22	0.074 (4)	0.070 (4)	0.049 (3)	−0.005 (3)	0.009 (3)	0.003 (3)
C23	0.074 (4)	0.046 (3)	0.053 (3)	0.014 (3)	0.006 (3)	0.006 (3)
C24	0.063 (4)	0.118 (6)	0.067 (4)	0.017 (4)	0.007 (3)	0.009 (4)
C25	0.040 (3)	0.051 (3)	0.058 (3)	−0.003 (2)	−0.004 (3)	−0.003 (3)
C26	0.049 (3)	0.048 (3)	0.058 (3)	−0.001 (3)	−0.014 (3)	−0.003 (3)
C27	0.065 (4)	0.055 (3)	0.045 (3)	0.002 (3)	−0.001 (3)	−0.003 (3)
C28	0.090 (5)	0.067 (4)	0.063 (4)	−0.002 (4)	0.027 (4)	0.003 (3)
C29	0.087 (5)	0.100 (6)	0.067 (4)	0.012 (4)	−0.004 (4)	−0.022 (4)
C30	0.065 (4)	0.047 (3)	0.072 (4)	−0.001 (3)	0.005 (3)	−0.006 (3)
C31	0.043 (3)	0.048 (3)	0.078 (4)	−0.002 (2)	−0.003 (3)	0.007 (3)
C32	0.081 (5)	0.104 (6)	0.108 (6)	0.040 (5)	−0.005 (5)	−0.005 (5)
C33	0.042 (3)	0.030 (2)	0.047 (3)	0.001 (2)	0.001 (2)	−0.010 (2)
C34	0.039 (3)	0.035 (2)	0.045 (3)	0.000 (2)	0.005 (2)	−0.002 (2)
C35	0.041 (3)	0.033 (2)	0.055 (3)	0.001 (2)	0.002 (2)	0.005 (2)
C36	0.045 (3)	0.037 (3)	0.049 (3)	−0.004 (2)	0.001 (2)	0.000 (2)

### Geometric parameters (Å, °)

**Table:**

Ni1—O1	2.046 (3)	C8—H8A	0.9600
Ni1—N4	2.094 (4)	C8—H8B	0.9600
Ni1—N2	2.110 (4)	C8—H8C	0.9600
Ni1—O3	2.142 (3)	C9—C10	1.505 (8)
Ni1—N1	2.174 (4)	C9—H9A	0.9700
Ni1—N3	2.173 (4)	C9—H9B	0.9700
Ni2—N7	2.087 (4)	C10—H10A	0.9700
Ni2—N5	2.090 (4)	C10—H10B	0.9700
Ni2—N6	2.117 (5)	C11—C13	1.508 (8)
Ni2—N8	2.122 (5)	C11—C12	1.519 (8)
Ni2—O5	2.174 (4)	C11—H11A	0.9800
Ni2—O4	2.203 (4)	C12—H12A	0.9600
Ni2—C36	2.516 (5)	C12—H12B	0.9600
Cl1—O8	1.405 (6)	C12—H12C	0.9600
Cl1—O7	1.405 (6)	C13—C14	1.524 (9)
Cl1—O9	1.411 (5)	C13—H13A	0.9700
Cl1—O6	1.414 (6)	C13—H13B	0.9700
Cl2—O13	1.416 (10)	C14—C16	1.531 (9)
Cl2—O11	1.435 (9)	C14—C15	1.570 (9)
Cl2—O12	1.415 (10)	C15—H15A	0.9600
Cl2—O10	1.463 (10)	C15—H15B	0.9600
Cl2'—O12'	1.446 (12)	C15—H15C	0.9600
Cl2'—O11'	1.424 (11)	C16—H16A	0.9600
Cl2'—O10'	1.443 (12)	C16—H16B	0.9600
Cl2'—O13'	1.439 (12)	C16—H16C	0.9600
O1—C33	1.250 (6)	C17—C18	1.481 (10)
O2—C33	1.234 (6)	C17—H17A	0.9700
O3—C34	1.438 (6)	C17—H17B	0.9700
O3—H3O	0.8400	C18—H18A	0.9700
O4—C36	1.270 (6)	C18—H18B	0.9700
O5—C36	1.257 (6)	C19—C22	1.523 (9)
O1W—H11	0.86 (9)	C19—C21	1.522 (10)
O1W—H12	0.86 (9)	C19—C20	1.540 (10)
N1—C1	1.489 (7)	C20—H20A	0.9600
N1—C14	1.506 (7)	C20—H20B	0.9600
N1—H1	0.85 (5)	C20—H20C	0.9600
N2—C2	1.471 (7)	C21—H21A	0.9600
N2—C3	1.487 (7)	C21—H21B	0.9600
N2—H2	0.84 (5)	C21—H21C	0.9600
N3—C9	1.480 (6)	C22—C23	1.517 (8)
N3—C6	1.513 (7)	C22—H22A	0.9700
N3—H3	0.88 (6)	C22—H22B	0.9700
N4—C10	1.472 (7)	C23—C24	1.531 (9)
N4—C11	1.511 (7)	C23—H23	0.9800
N4—H4	0.85 (5)	C24—H24A	0.9600
N5—C31	1.471 (8)	C24—H24B	0.9600
N5—C17	1.494 (8)	C24—H24C	0.9600
N5—H5	0.85 (5)	C25—C26	1.487 (8)
N6—C18	1.496 (8)	C25—H25A	0.9700
N6—C19	1.510 (9)	C25—H25B	0.9700
N6—H6	0.85 (5)	C26—H26A	0.9700
N7—C23	1.478 (7)	C26—H26B	0.9700
N7—C25	1.483 (7)	C27—C28	1.508 (9)
N7—H7	0.85 (2)	C27—C30	1.534 (8)
N8—C26	1.478 (7)	C27—C29	1.547 (9)
N8—C27	1.491 (7)	C28—H28A	0.9600
N8—H8	0.85 (5)	C28—H28B	0.9600
C1—C2	1.501 (8)	C28—H28C	0.9600
C1—H1A	0.9700	C29—H29A	0.9600
C1—H1B	0.9700	C29—H29B	0.9600
C2—H2A	0.9700	C29—H29C	0.9600
C2—H2B	0.9700	C30—C31	1.514 (9)
C3—C4	1.518 (7)	C30—H30A	0.9700
C3—C5	1.512 (7)	C30—H30B	0.9700
C3—H3a	0.9800	C31—C32	1.529 (9)
C4—H4A	0.9600	C31—H31	0.9800
C4—H4B	0.9600	C32—H32A	0.9600
C4—H4C	0.9600	C32—H32B	0.9600
C5—C6	1.548 (8)	C32—H32C	0.9600
C5—H5A	0.9700	C33—C34	1.536 (7)
C5—H5B	0.9700	C34—C35	1.507 (7)
C6—C7	1.484 (8)	C34—H34	0.9800
C6—C8	1.548 (8)	C35—C36	1.510 (7)
C7—H7A	0.9600	C35—H35A	0.9700
C7—H7B	0.9600	C35—H35B	0.9700
C7—H7C	0.9600		
			
O1—Ni1—N4	89.28 (15)	C10—C9—H9B	109.8
O1—Ni1—N2	166.91 (15)	H9A—C9—H9B	108.2
N4—Ni1—N2	103.22 (17)	N4—C10—C9	109.8 (4)
O1—Ni1—O3	76.30 (13)	N4—C10—H10A	109.7
N4—Ni1—O3	164.72 (15)	C9—C10—H10A	109.7
N2—Ni1—O3	91.55 (15)	N4—C10—H10B	109.7
O1—Ni1—N1	99.79 (15)	C9—C10—H10B	109.7
N4—Ni1—N1	89.60 (17)	H10A—C10—H10B	108.2
N2—Ni1—N1	84.37 (17)	C13—C11—N4	110.4 (4)
O3—Ni1—N1	87.94 (16)	C13—C11—C12	110.9 (5)
O1—Ni1—N3	88.08 (15)	N4—C11—C12	111.5 (5)
N4—Ni1—N3	84.36 (17)	C13—C11—H11A	108.0
N2—Ni1—N3	89.29 (17)	N4—C11—H11A	108.0
O3—Ni1—N3	99.94 (15)	C12—C11—H11A	108.0
N1—Ni1—N3	170.03 (16)	C11—C12—H12A	109.5
N7—Ni2—N5	104.33 (16)	C11—C12—H12B	109.5
N7—Ni2—N6	90.01 (18)	H12A—C12—H12B	109.5
N5—Ni2—N6	85.9 (2)	C11—C12—H12C	109.5
N7—Ni2—N8	85.12 (18)	H12A—C12—H12C	109.5
N5—Ni2—N8	90.06 (18)	H12B—C12—H12C	109.5
N6—Ni2—N8	172.74 (18)	C11—C13—C14	120.3 (5)
N7—Ni2—O5	100.08 (15)	C11—C13—H13A	107.2
N5—Ni2—O5	155.28 (16)	C14—C13—H13A	107.2
N6—Ni2—O5	98.00 (17)	C11—C13—H13B	107.2
N8—Ni2—O5	88.17 (16)	C14—C13—H13B	107.2
N7—Ni2—O4	158.72 (15)	H13A—C13—H13B	106.9
N5—Ni2—O4	96.04 (15)	N1—C14—C13	110.1 (4)
N6—Ni2—O4	85.28 (16)	N1—C14—C16	107.4 (5)
N8—Ni2—O4	101.17 (16)	C13—C14—C16	111.8 (5)
O5—Ni2—O4	60.25 (13)	N1—C14—C15	111.4 (5)
N7—Ni2—C36	129.40 (16)	C13—C14—C15	107.8 (5)
N5—Ni2—C36	126.19 (17)	C16—C14—C15	108.2 (5)
N6—Ni2—C36	90.80 (17)	C14—C15—H15A	109.5
N8—Ni2—C36	96.45 (17)	C14—C15—H15B	109.5
O5—Ni2—C36	29.99 (15)	H15A—C15—H15B	109.5
O4—Ni2—C36	30.30 (15)	C14—C15—H15C	109.5
O8—Cl1—O7	110.0 (4)	H15A—C15—H15C	109.5
O8—Cl1—O9	111.4 (4)	H15B—C15—H15C	109.5
O7—Cl1—O9	109.9 (4)	C14—C16—H16A	109.5
O8—Cl1—O6	108.5 (4)	C14—C16—H16B	109.5
O7—Cl1—O6	107.2 (4)	H16A—C16—H16B	109.5
O9—Cl1—O6	109.7 (4)	C14—C16—H16C	109.5
O13—Cl2—O11	111.1 (11)	H16A—C16—H16C	109.5
O13—Cl2—O12	113.1 (11)	H16B—C16—H16C	109.5
O11—Cl2—O12	108.8 (8)	C18—C17—N5	109.8 (5)
O13—Cl2—O10	110.5 (11)	C18—C17—H17A	109.7
O11—Cl2—O10	113.5 (8)	N5—C17—H17A	109.7
O12—Cl2—O10	99.4 (9)	C18—C17—H17B	109.7
O12'—Cl2'—O11'	109.9 (16)	N5—C17—H17B	109.7
O12'—Cl2'—O10'	108.9 (16)	H17A—C17—H17B	108.2
O11'—Cl2'—O10'	114.5 (16)	C17—C18—N6	109.9 (5)
O12'—Cl2'—O13'	106.2 (15)	C17—C18—H18A	109.7
O11'—Cl2'—O13'	108.1 (13)	N6—C18—H18A	109.7
O10'—Cl2'—O13'	108.9 (16)	C17—C18—H18B	109.7
C33—O1—Ni1	121.1 (3)	N6—C18—H18B	109.7
C34—O3—Ni1	115.8 (3)	H18A—C18—H18B	108.2
C34—O3—H3O	122.1	N6—C19—C22	110.3 (5)
Ni1—O3—H3O	122.1	N6—C19—C21	108.1 (6)
C36—O4—Ni2	88.6 (3)	C22—C19—C21	111.9 (7)
C36—O5—Ni2	90.2 (3)	N6—C19—C20	111.6 (7)
H11—O1W—H12	103 (13)	C22—C19—C20	107.7 (6)
C1—N1—C14	113.7 (4)	C21—C19—C20	107.2 (6)
C1—N1—Ni1	103.2 (3)	C19—C20—H20A	109.5
C14—N1—Ni1	121.2 (3)	C19—C20—H20B	109.5
C1—N1—H1	109 (5)	H20A—C20—H20B	109.5
C14—N1—H1	107 (5)	C19—C20—H20C	109.5
Ni1—N1—H1	101 (5)	H20A—C20—H20C	109.5
C2—N2—C3	113.5 (4)	H20B—C20—H20C	109.5
C2—N2—Ni1	104.9 (3)	C19—C21—H21A	109.5
C3—N2—Ni1	115.6 (3)	C19—C21—H21B	109.5
C2—N2—H2	109 (4)	H21A—C21—H21B	109.5
C3—N2—H2	105 (5)	C19—C21—H21C	109.5
Ni1—N2—H2	109 (5)	H21A—C21—H21C	109.5
C9—N3—C6	113.8 (4)	H21B—C21—H21C	109.5
C9—N3—Ni1	104.2 (3)	C23—C22—C19	120.1 (5)
C6—N3—Ni1	122.1 (3)	C23—C22—H22A	107.3
C9—N3—H3	106 (4)	C19—C22—H22A	107.3
C6—N3—H3	101 (4)	C23—C22—H22B	107.3
Ni1—N3—H3	109 (4)	C19—C22—H22B	107.3
C10—N4—C11	111.9 (4)	H22A—C22—H22B	106.9
C10—N4—Ni1	104.2 (3)	N7—C23—C22	111.4 (5)
C11—N4—Ni1	115.9 (3)	N7—C23—C24	111.3 (5)
C10—N4—H4	112 (4)	C22—C23—C24	108.6 (5)
C11—N4—H4	108 (4)	N7—C23—H23	108.5
Ni1—N4—H4	105 (4)	C22—C23—H23	108.5
C31—N5—C17	113.7 (5)	C24—C23—H23	108.5
C31—N5—Ni2	116.4 (3)	C23—C24—H24A	109.5
C17—N5—Ni2	103.8 (4)	C23—C24—H24B	109.5
C31—N5—H5	112 (5)	H24A—C24—H24B	109.5
C17—N5—H5	102 (5)	C23—C24—H24C	109.5
Ni2—N5—H5	108 (5)	H24A—C24—H24C	109.5
C18—N6—C19	115.0 (5)	H24B—C24—H24C	109.5
C18—N6—Ni2	103.5 (4)	N7—C25—C26	109.9 (4)
C19—N6—Ni2	120.7 (4)	N7—C25—H25A	109.7
C18—N6—H6	104 (5)	C26—C25—H25A	109.7
C19—N6—H6	103 (5)	N7—C25—H25B	109.7
Ni2—N6—H6	110 (5)	C26—C25—H25B	109.7
C23—N7—C25	112.9 (4)	H25A—C25—H25B	108.2
C23—N7—Ni2	119.4 (3)	N8—C26—C25	109.5 (4)
C25—N7—Ni2	104.0 (3)	N8—C26—H26A	109.8
C23—N7—H7	109 (4)	C25—C26—H26A	109.8
C25—N7—H7	101 (4)	N8—C26—H26B	109.8
Ni2—N7—H7	108 (4)	C25—C26—H26B	109.8
C26—N8—C27	115.0 (4)	H26A—C26—H26B	108.2
C26—N8—Ni2	104.6 (3)	N8—C27—C28	107.9 (5)
C27—N8—Ni2	120.8 (3)	N8—C27—C30	110.9 (4)
C26—N8—H8	109 (5)	C28—C27—C30	111.0 (5)
C27—N8—H8	110 (5)	N8—C27—C29	111.5 (5)
Ni2—N8—H8	95 (4)	C28—C27—C29	107.9 (5)
N1—C1—C2	109.8 (5)	C30—C27—C29	107.6 (5)
N1—C1—H1A	109.7	C27—C28—H28A	109.5
C2—C1—H1A	109.7	C27—C28—H28B	109.5
N1—C1—H1B	109.7	H28A—C28—H28B	109.5
C2—C1—H1B	109.7	C27—C28—H28C	109.5
H1A—C1—H1B	108.2	H28A—C28—H28C	109.5
N2—C2—C1	109.7 (4)	H28B—C28—H28C	109.5
N2—C2—H2A	109.7	C27—C29—H29A	109.5
C1—C2—H2A	109.7	C27—C29—H29B	109.5
N2—C2—H2B	109.7	H29A—C29—H29B	109.5
C1—C2—H2B	109.7	C27—C29—H29C	109.5
H2A—C2—H2B	108.2	H29A—C29—H29C	109.5
N2—C3—C4	113.1 (5)	H29B—C29—H29C	109.5
N2—C3—C5	110.2 (4)	C31—C30—C27	118.5 (5)
C4—C3—C5	110.7 (5)	C31—C30—H30A	107.7
N2—C3—H3a	107.5	C27—C30—H30A	107.7
C4—C3—H3a	107.5	C31—C30—H30B	107.7
C5—C3—H3a	107.5	C27—C30—H30B	107.7
C3—C4—H4A	109.5	H30A—C30—H30B	107.1
C3—C4—H4B	109.5	N5—C31—C30	110.8 (5)
H4A—C4—H4B	109.5	N5—C31—C32	112.6 (6)
C3—C4—H4C	109.5	C30—C31—C32	108.8 (6)
H4A—C4—H4C	109.5	N5—C31—H31	108.2
H4B—C4—H4C	109.5	C30—C31—H31	108.2
C3—C5—C6	119.3 (4)	C32—C31—H31	108.2
C3—C5—H5A	107.5	C31—C32—H32A	109.5
C6—C5—H5A	107.5	C31—C32—H32B	109.5
C3—C5—H5B	107.5	H32A—C32—H32B	109.5
C6—C5—H5B	107.5	C31—C32—H32C	109.5
H5A—C5—H5B	107.0	H32A—C32—H32C	109.5
C7—C6—N3	108.3 (4)	H32B—C32—H32C	109.5
C7—C6—C8	108.2 (5)	O2—C33—O1	126.6 (5)
N3—C6—C8	111.2 (5)	O2—C33—C34	115.3 (4)
C7—C6—C5	111.4 (5)	O1—C33—C34	118.1 (4)
N3—C6—C5	110.4 (4)	O3—C34—C35	111.7 (4)
C8—C6—C5	107.2 (5)	O3—C34—C33	108.2 (4)
C6—C7—H7A	109.5	C35—C34—C33	112.0 (4)
C6—C7—H7B	109.5	O3—C34—H34	108.2
H7A—C7—H7B	109.5	C35—C34—H34	108.2
C6—C7—H7C	109.5	C33—C34—H34	108.2
H7A—C7—H7C	109.5	C34—C35—C36	113.2 (4)
H7B—C7—H7C	109.5	C34—C35—H35A	108.9
C6—C8—H8A	109.5	C36—C35—H35A	108.9
C6—C8—H8B	109.5	C34—C35—H35B	108.9
H8A—C8—H8B	109.5	C36—C35—H35B	108.9
C6—C8—H8C	109.5	H35A—C35—H35B	107.7
H8A—C8—H8C	109.5	O5—C36—O4	120.8 (5)
H8B—C8—H8C	109.5	O5—C36—C35	120.5 (5)
N3—C9—C10	109.4 (4)	O4—C36—C35	118.8 (4)
N3—C9—H9A	109.8	O5—C36—Ni2	59.8 (3)
C10—C9—H9A	109.8	O4—C36—Ni2	61.1 (2)
N3—C9—H9B	109.8	C35—C36—Ni2	175.7 (4)
			
N4—Ni1—O1—C33	170.1 (4)	Ni1—N2—C2—C1	44.9 (5)
N2—Ni1—O1—C33	−27.0 (9)	N1—C1—C2—N2	−61.4 (6)
O3—Ni1—O1—C33	−4.8 (4)	C2—N2—C3—C4	47.5 (7)
N1—Ni1—O1—C33	80.6 (4)	Ni1—N2—C3—C4	168.7 (4)
N3—Ni1—O1—C33	−105.5 (4)	C2—N2—C3—C5	171.9 (4)
O1—Ni1—O3—C34	6.3 (3)	Ni1—N2—C3—C5	−66.8 (5)
N4—Ni1—O3—C34	−13.4 (8)	N2—C3—C5—C6	74.0 (6)
N2—Ni1—O3—C34	−178.7 (3)	C4—C3—C5—C6	−160.1 (5)
N1—Ni1—O3—C34	−94.3 (3)	C9—N3—C6—C7	157.0 (5)
N3—Ni1—O3—C34	91.8 (3)	Ni1—N3—C6—C7	−76.7 (5)
N7—Ni2—O4—C36	21.9 (6)	C9—N3—C6—C8	38.2 (6)
N5—Ni2—O4—C36	−174.9 (3)	Ni1—N3—C6—C8	164.5 (4)
N6—Ni2—O4—C36	99.7 (3)	C9—N3—C6—C5	−80.7 (5)
N8—Ni2—O4—C36	−83.6 (3)	Ni1—N3—C6—C5	45.5 (5)
O5—Ni2—O4—C36	−2.3 (3)	C3—C5—C6—C7	59.7 (7)
N7—Ni2—O5—C36	−169.0 (3)	C3—C5—C6—N3	−60.8 (6)
N5—Ni2—O5—C36	20.1 (6)	C3—C5—C6—C8	177.9 (5)
N6—Ni2—O5—C36	−77.6 (3)	C6—N3—C9—C10	173.3 (4)
N8—Ni2—O5—C36	106.3 (3)	Ni1—N3—C9—C10	38.1 (5)
O4—Ni2—O5—C36	2.3 (3)	C11—N4—C10—C9	173.7 (4)
O1—Ni1—N1—C1	179.2 (3)	Ni1—N4—C10—C9	47.8 (4)
N4—Ni1—N1—C1	90.0 (4)	N3—C9—C10—N4	−60.8 (5)
N2—Ni1—N1—C1	−13.3 (3)	C10—N4—C11—C13	176.8 (4)
O3—Ni1—N1—C1	−105.1 (4)	Ni1—N4—C11—C13	−63.9 (5)
O1—Ni1—N1—C14	50.5 (4)	C10—N4—C11—C12	53.1 (6)
N4—Ni1—N1—C14	−38.7 (4)	Ni1—N4—C11—C12	172.4 (4)
N2—Ni1—N1—C14	−142.0 (4)	N4—C11—C13—C14	72.6 (6)
O3—Ni1—N1—C14	126.3 (4)	C12—C11—C13—C14	−163.4 (5)
O1—Ni1—N2—C2	92.6 (7)	C1—N1—C14—C13	−76.0 (6)
N4—Ni1—N2—C2	−104.9 (4)	Ni1—N1—C14—C13	47.9 (6)
O3—Ni1—N2—C2	71.1 (4)	C1—N1—C14—C16	162.0 (5)
N1—Ni1—N2—C2	−16.7 (4)	Ni1—N1—C14—C16	−74.1 (5)
N3—Ni1—N2—C2	171.0 (4)	C1—N1—C14—C15	43.6 (7)
O1—Ni1—N2—C3	−33.1 (9)	Ni1—N1—C14—C15	167.5 (4)
N4—Ni1—N2—C3	129.3 (4)	C11—C13—C14—N1	−62.5 (7)
O3—Ni1—N2—C3	−54.7 (4)	C11—C13—C14—C16	56.9 (7)
N1—Ni1—N2—C3	−142.4 (4)	C11—C13—C14—C15	175.7 (5)
N3—Ni1—N2—C3	45.3 (4)	C31—N5—C17—C18	−171.1 (5)
O1—Ni1—N3—C9	−99.3 (3)	Ni2—N5—C17—C18	−43.8 (5)
N4—Ni1—N3—C9	−9.8 (3)	N5—C17—C18—N6	60.1 (7)
N2—Ni1—N3—C9	93.5 (3)	C19—N6—C18—C17	−174.9 (5)
O3—Ni1—N3—C9	−175.0 (3)	Ni2—N6—C18—C17	−41.2 (5)
O1—Ni1—N3—C6	130.3 (4)	C18—N6—C19—C22	73.4 (7)
N4—Ni1—N3—C6	−140.3 (4)	Ni2—N6—C19—C22	−51.8 (6)
N2—Ni1—N3—C6	−36.9 (4)	C18—N6—C19—C21	−164.0 (6)
O3—Ni1—N3—C6	54.5 (4)	Ni2—N6—C19—C21	70.8 (7)
O1—Ni1—N4—C10	68.1 (3)	C18—N6—C19—C20	−46.3 (7)
N2—Ni1—N4—C10	−108.0 (3)	Ni2—N6—C19—C20	−171.5 (5)
O3—Ni1—N4—C10	87.2 (7)	N6—C19—C22—C23	63.5 (8)
N1—Ni1—N4—C10	167.9 (3)	C21—C19—C22—C23	−56.9 (8)
N3—Ni1—N4—C10	−20.1 (3)	C20—C19—C22—C23	−174.5 (7)
O1—Ni1—N4—C11	−55.4 (4)	C25—N7—C23—C22	179.9 (5)
N2—Ni1—N4—C11	128.6 (4)	Ni2—N7—C23—C22	57.2 (6)
O3—Ni1—N4—C11	−36.2 (8)	C25—N7—C23—C24	−58.8 (6)
N1—Ni1—N4—C11	44.5 (4)	Ni2—N7—C23—C24	178.6 (4)
N3—Ni1—N4—C11	−143.5 (4)	C19—C22—C23—N7	−67.2 (7)
N7—Ni2—N5—C31	−129.5 (4)	C19—C22—C23—C24	169.9 (6)
N6—Ni2—N5—C31	141.5 (4)	C23—N7—C25—C26	−175.7 (4)
N8—Ni2—N5—C31	−44.5 (4)	Ni2—N7—C25—C26	−44.9 (5)
O5—Ni2—N5—C31	41.2 (6)	C27—N8—C26—C25	−174.6 (4)
O4—Ni2—N5—C31	56.7 (4)	Ni2—N8—C26—C25	−39.8 (5)
C36—Ni2—N5—C31	53.5 (4)	N7—C25—C26—N8	59.7 (6)
N7—Ni2—N5—C17	104.8 (4)	C26—N8—C27—C28	−161.0 (5)
N6—Ni2—N5—C17	15.9 (4)	Ni2—N8—C27—C28	72.1 (5)
N8—Ni2—N5—C17	−170.2 (4)	C26—N8—C27—C30	77.3 (6)
O5—Ni2—N5—C17	−84.4 (5)	Ni2—N8—C27—C30	−49.7 (6)
O4—Ni2—N5—C17	−68.9 (4)	C26—N8—C27—C29	−42.6 (7)
C36—Ni2—N5—C17	−72.1 (4)	Ni2—N8—C27—C29	−169.5 (4)
N7—Ni2—N6—C18	−91.2 (4)	N8—C27—C30—C31	62.6 (7)
N5—Ni2—N6—C18	13.1 (4)	C28—C27—C30—C31	−57.4 (7)
O5—Ni2—N6—C18	168.6 (4)	C29—C27—C30—C31	−175.2 (6)
O4—Ni2—N6—C18	109.5 (4)	C17—N5—C31—C30	−176.0 (5)
C36—Ni2—N6—C18	139.4 (4)	Ni2—N5—C31—C30	63.5 (5)
N7—Ni2—N6—C19	39.1 (4)	C17—N5—C31—C32	−53.9 (7)
N5—Ni2—N6—C19	143.5 (4)	Ni2—N5—C31—C32	−174.4 (5)
O5—Ni2—N6—C19	−61.1 (4)	C27—C30—C31—N5	−71.3 (7)
O4—Ni2—N6—C19	−120.1 (4)	C27—C30—C31—C32	164.3 (6)
C36—Ni2—N6—C19	−90.3 (4)	Ni1—O1—C33—O2	−177.9 (4)
N5—Ni2—N7—C23	−127.0 (4)	Ni1—O1—C33—C34	2.5 (6)
N6—Ni2—N7—C23	−41.2 (4)	Ni1—O3—C34—C35	−130.4 (3)
N8—Ni2—N7—C23	144.2 (4)	Ni1—O3—C34—C33	−6.6 (5)
O5—Ni2—N7—C23	57.0 (4)	O2—C33—C34—O3	−176.7 (4)
O4—Ni2—N7—C23	35.8 (7)	O1—C33—C34—O3	2.9 (6)
C36—Ni2—N7—C23	49.9 (4)	O2—C33—C34—C35	−53.1 (6)
N5—Ni2—N7—C25	106.1 (3)	O1—C33—C34—C35	126.5 (5)
N6—Ni2—N7—C25	−168.1 (3)	O3—C34—C35—C36	−57.3 (5)
N8—Ni2—N7—C25	17.3 (3)	C33—C34—C35—C36	−178.9 (4)
O5—Ni2—N7—C25	−69.9 (3)	Ni2—O5—C36—O4	−4.0 (5)
O4—Ni2—N7—C25	−91.1 (5)	Ni2—O5—C36—C35	175.0 (4)
C36—Ni2—N7—C25	−77.0 (4)	Ni2—O4—C36—O5	4.0 (5)
N7—Ni2—N8—C26	11.9 (3)	Ni2—O4—C36—C35	−175.0 (4)
N5—Ni2—N8—C26	−92.5 (3)	C34—C35—C36—O5	−144.5 (5)
O5—Ni2—N8—C26	112.2 (3)	C34—C35—C36—O4	34.5 (7)
O4—Ni2—N8—C26	171.3 (3)	N7—Ni2—C36—O5	14.0 (4)
C36—Ni2—N8—C26	141.0 (3)	N5—Ni2—C36—O5	−169.7 (3)
N7—Ni2—N8—C27	143.4 (4)	N6—Ni2—C36—O5	104.7 (3)
N5—Ni2—N8—C27	39.1 (4)	N8—Ni2—C36—O5	−74.9 (3)
O5—Ni2—N8—C27	−116.3 (4)	O4—Ni2—C36—O5	−176.1 (5)
O4—Ni2—N8—C27	−57.1 (4)	N7—Ni2—C36—O4	−169.9 (3)
C36—Ni2—N8—C27	−87.4 (4)	N5—Ni2—C36—O4	6.3 (4)
C14—N1—C1—C2	174.7 (5)	N6—Ni2—C36—O4	−79.2 (3)
Ni1—N1—C1—C2	41.5 (5)	N8—Ni2—C36—O4	101.1 (3)
C3—N2—C2—C1	172.0 (5)	O5—Ni2—C36—O4	176.1 (5)

### Hydrogen-bond geometry (Å, °)

**Table:**

*D*—H···*A*	*D*—H	H···*A*	*D*···*A*	*D*—H···*A*
N2—H2···O6^i^	0.84 (5)	2.41 (2)	3.230 (7)	165 (6)
N5—H5···O2^ii^	0.85 (5)	2.14 (3)	2.940 (6)	158 (6)
N7—H7···O2^ii^	0.85 (2)	2.06 (2)	2.869 (5)	160 (6)
O1w—H11···O5	0.86 (9)	2.09 (7)	2.862 (9)	149 (12)
O1w—H12···O6	0.86 (9)	2.05 (8)	2.784 (12)	143 (13)

Symmetry codes: (i) *x*−1, *y*, *z*; (ii) −*x*+1, *y*+1/2, −*z*+3/2.

## References

[bb1] Barbour, L. J. (2001). *J. Supramol. Chem.* **1**, 189–211.

[bb2] Bruker (1999). *SMART* and *SAINT-Plus* Bruker AXS Inc, Madison, Wisconsin, USA.

[bb3] Flack, H. D. (1983). *Acta Cryst.* A**39**, 876–881.

[bb4] Ou, G.-C., Zhang, M. & Yuan, X.-Y. (2009). *Acta Cryst.* E**65**, m726. 10.1107/S1600536809020169 PMC2969201 21582669

[bb5] Sheldrick, G. M. (1996). *SADABS* University of Göttingen, Germany.

[bb6] Sheldrick, G. M. (2008). *Acta Cryst.* A**64**, 112–122. 10.1107/S0108767307043930 18156677

[bb7] Westrip, S. P. (2009). *publCIF* In preparation.

